# Genotyping and Antimicrobial Susceptibility of *Clostridium perfringens* and *Clostridioides difficile* in Camel Minced Meat

**DOI:** 10.3390/pathogens10121640

**Published:** 2021-12-19

**Authors:** Mahmoud Fayez, Waleed R. El-Ghareeb, Ahmed Elmoslemany, Saleem J. Alsunaini, Mohamed Alkafafy, Othman M. Alzahrani, Samy F. Mahmoud, Ibrahim Elsohaby

**Affiliations:** 1 Al-Ahsa Veterinary Diagnostic Lab, Ministry of Environment, Water and Agriculture, Al-Ahsa 31982, Saudi Arabia; mahmoudfayez30@hotmail.com (M.F.); sslam0002@gmail.com (S.J.A.); 2 Department of Bacteriology, Veterinary Serum and Vaccine Research Institute, Ministry of Agriculture, Cairo 11381, Egypt; 3 Department of Veterinary Public Health and Animal Husbandry, College of Veterinary Medicine, King Faisal University, P.O. Box 400, Al-Ahsa 31982, Saudi Arabia; welsaid@kfu.edu.sa; 4 Hygiene and Preventive Medicine Department, Faculty of Veterinary Medicine, Kafrelsheikh University, Kafr El-Sheikh 33516, Egypt; aelmoslemany@gmail.com; 5 Department of Biotechnology, College of Science, Taif University, P.O. Box 11099, Taif 21944, Saudi Arabia; m.kafafy@tu.edu.sa (M.A.); s.farouk@tu.edu.sa (S.F.M.); 6 Department of Biology, College of Science, Taif University, P.O. Box 11099, Taif 21944, Saudi Arabia; o.alzahrani@tu.edu.sa; 7 Department of Animal Medicine, Faculty of Veterinary Medicine, Zagazig University, Zagazig 44511, Egypt; 8 Department of Health Management, Atlantic Veterinary College, University of Prince Edward Island, Charlottetown, PE C1A 4P3, Canada; 9 Department of Infectious Diseases and Public Health, Jockey Club of Veterinary Medicine and Life Sciences, City University of Hong Kong, Kowloon, Hong Kong

**Keywords:** *C. perfringens*, *C. difficile*, minced meat, camel, toxin genes, genotypes

## Abstract

The present study aimed to determine the occurrence, genotypes, and antimicrobial resistance of *Clostridium perfringens* (*C. perfringens*) and *Clostridioides difficile* (*C. difficile*) in camel minced meat samples collected from small butcher shops and supermarkets in Al-Ahsa Governorate, Saudi Arabia. A total of 100 camel minced meat samples were randomly collected from small butcher’s shops (n = 50) and supermarkets (n = 50) in Al-Ahsa Governorate, Saudi Arabia. *C. perfringens* and *C. difficile* were isolated and identified using the VITEK-2 compact system and 16S rRNA gene amplification. Genotypes, toxin genes, and antimicrobial susceptibility of the isolates were determined. Moreover, ELISA was used to detect *C. perfringens* and *C. difficile* toxins. *C. perfringens* and *C. difficile* were isolated from 14% and 4% of the tested minced meat samples, respectively. Out of the 14 *C. perfringens* isolates, type A (64.3%), type B (7.1%), type C (21.5%), and type D (7.1%) were detected. However, out of the four *C. difficile* isolates, three (75%) were type A^+^B^+^ and one (25%) was type A^−^B^+^. None of the *C. perfringens* or *C. difficile* toxins were identified using ELISA. *C. perfringens* and *C. difficile* isolates exhibited a high rate of resistance to tetracycline (56% and 75%, respectively). However, all isolates were susceptible to amoxicillin-clavulanate. Multidrug resistance was observed in three (21.4%) *C. perfringens* and one (25%) *C. difficile* isolates. In conclusion, camel minced meat was contaminated with *C. perfringens* and *C. difficile*, which present a potential risk of food poisoning. The majority of the isolates were resistant to at least one antimicrobial, and some isolates were multidrug-resistant. Therefore, food safety standards and frequent inspections of abattoirs, small butcher shops, and supermarkets should be enforced.

## 1. Introduction

Food hygiene is described as all the conditions and measures necessary to ensure that food is safe and fit for human consumption during production, processing, storage, distribution, and preparation [1]. Foodborne diseases are caused by food contamination and can occur at any stage of the food production, delivery, and consumption chain. There are over 200 foodborne hazards, including microbiological hazards such as bacteria, viruses, parasites, and chemical contaminants, that arise naturally or due to pollution, food processing, packaging, transportation, or storage [2]. Foodborne illnesses and outbreaks are among the leading causes of death globally [3,4]. According to studies, the magnitude of foodborne illnesses in 2010 included 600 million illnesses and 420,000 deaths worldwide [5,6]. Nausea, vomiting, retching, diarrhea, abdominal discomfort, prostration, abdominal cramps, fever, chills, headache, and arthralgia are just a few of the symptoms [7].

*Clostridia* spp. are anaerobic Gram-positive, spore-forming and non-motile bacteria commonly found in the intestinal tract of humans and animals and the soil [8]. *Clostridium perfringens* (*C. perfringens*) is the most widespread species and among the most common foodborne pathogens in industrial countries. There are several toxigenic types of *C. perfringens*, including A, B, C, D, and E. However, type A is primarily associated with foodborne illness [9]. *C. perfringens* food poisoning can occur when cooked meat is not adequately heated or refrigerated before serving. Illness often arises 8–15 h after consuming contaminated food. The symptoms include strong abdominal cramps, gas, and diarrhea (nausea and infrequent vomiting) [10]. *Clostridioides difficile* (*C. difficile*) is also associated with food poisoning, with symptoms ranging from mild diarrhea to life-threatening pseudomembranous colitis [11,12].

The recent rise and re-emergence of antimicrobial-resistant foodborne bacteria necessitate coordinated efforts, particularly in developing countries [13]. Antibacterial resistance can spread through the food chain, either directly or indirectly. Direct exposure happens when a human comes into contact with an animal or its blood, saliva, milk, sperm, feces, or urine. Indirect contact occurs, following consumption of infected food products such as eggs, meat, and dairy products [14,15].

Camel is a versatile animal that is used for milk, meat, wool, transportation, racing, tourism, agricultural work, and beauty contests. Camel meat is a major source of animal protein in many African and Asian countries, particularly in locations where the environment makes it difficult for other animals to produce. Camel meat is healthier than other meat animals since the carcass has less fat and has lower cholesterol levels in the fat. Camel meat also has a higher proportion of polyunsaturated fatty acids than beef [16,17].

In Saudi Arabia, a number of surveys have investigated the prevalence of camel meat contamination with some aerobic bacteria such as *Staphylococcus aureus*, *E. coli*, and *Salmonella* [18,19,20,21]. To the best of the authors’ knowledge, no literature exists that investigates the anaerobic contamination of camel meat with bacteria. Therefore, the main goal of this study is to determine the occurrence, genotypes and antimicrobial resistance of *C. perfringens* and *C. difficile* in camel minced meat samples collected from small butcher shops and supermarkets in Al-Ahsa Governorate, Saudi Arabia. 

## 2. Results

### 2.1. Isolation and Identification of C. perfringens and C. difficile

Out of the 100 minced meat samples tested in the present study, 14% and 4% were contaminated with *C. perfringens* and *C. difficile*, respectively. More than 70% of the *C. perfringens* and *C. difficile* isolates were recovered from samples collected from butcher shops (Table 1). 

**Figure 1:**
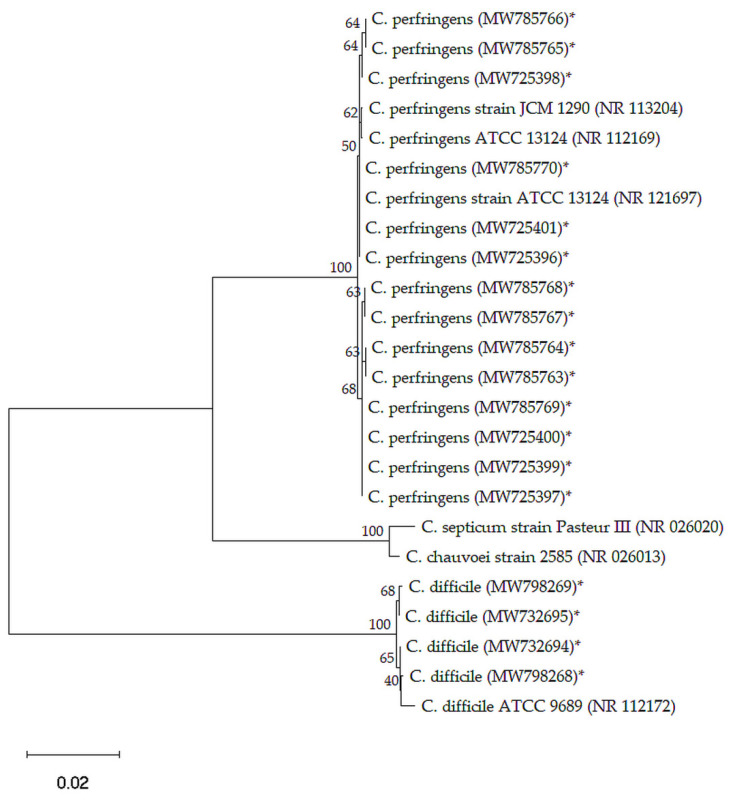
Phylogenetic tree based on 16S rRNA sequences of *C. perfringens* and *C. difficile* isolates recovered from camel minced meat samples. The asterisk (*) refers to *C. perfringens* and *C. difficile* isolates recovered in this study.

The number of *C. perfringens* in positive samples ranged from 200 to 2 × 10^3^ CFU/g. Based on the 16S rRNA sequence analysis, the 14 *C. perfringens* and four *C. difficile* isolates were clustered with the reference *C. perfringens* (NR 121697, NR 113204, and NR 112169) and *C. difficile* (NR 112172), respectively, with a similarity level of 100% (Figure 1).

### 2.2. Genotyping of C. perfringens and C. difficile Isolates 

**Figure 2:**
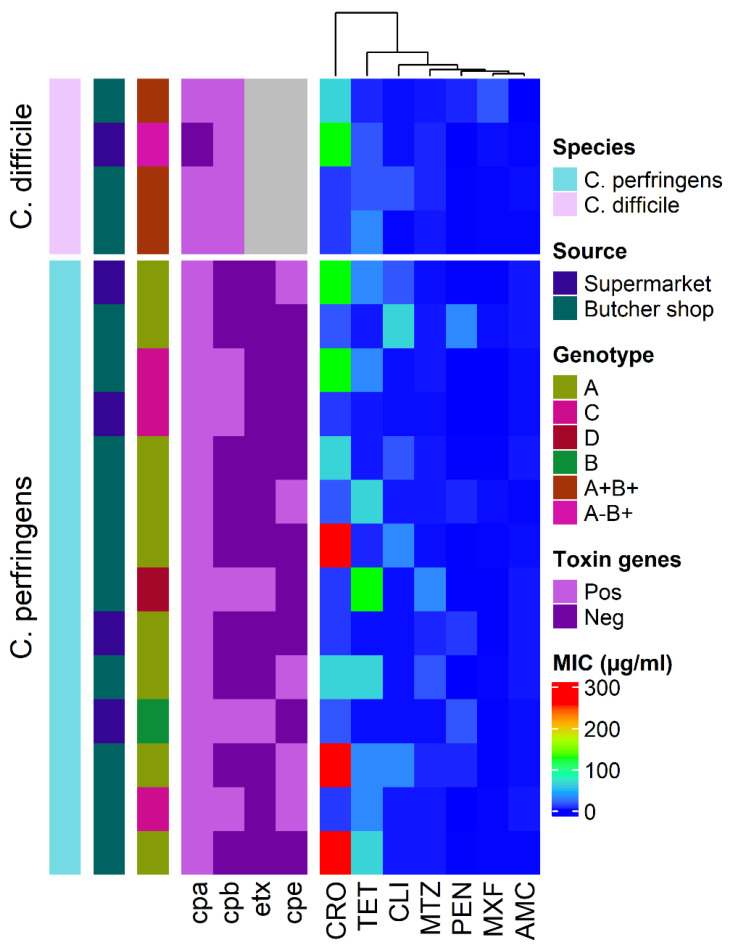
Heat map of the toxin genes and antimicrobial resistance profiles of *C. perfringens* and *C. difficile* genotypes recovered from camel minced meat collected from small butcher shops and supermarkets. Antimicrobials are ceftriaxone (CRO), tetracycline (TET), clindamycin (CLI), metronidazole (MTZ), penicillin (PEN), moxifloxacin (MXF), and amoxicillin-clavulanate (AMC).

PCR genotyping revealed that *C. perfringens* isolated from camel minced meat was related to type A, B, C, and D, whereas *C. difficile* isolates were related to type A^+^B^+^ and A^−^B^+^ (Figure 2). 

Table 2 shows the distribution of *C. perfringens* and *C. difficile* types and toxin genes in camel minced meat samples. Out of the 14 *C. perfringens* isolates, nine (64.3%) were type A, one (7.1%) was type B, three (21.5%) were type C, and one (7.1%) was type D. *C. perfringens* type A with only *cpa^+^* gene was found in five (55.6%) isolates and *cpa^+^* associated *cpe^+^* genes were found in four (44.4%) isolates. However, out of the four *C. difficile* isolates, three (75%) were type A^+^B^+^ and one (25%) was type A^−^B^+^.

### 2.3. Detection of C. perfringens and C. difficile Toxin by ELISA

None of the *C. perfringens* or *C. difficile* toxins were detected in the supernatant of homogenized meat samples using ELISA. The alpha toxin of *C. perfringens* was detected by ELISA in culture supernatants from all *C. perfringens* isolates, but the beta and epsilon toxins were detected in culture supernatants from four and one *C. perfringens* isolates, respectively. *C. difficile* alpha and beta toxins were detected in the culture supernatant of three isolates, but only beta toxin was detected in one isolate’s culture supernatant. Enterotoxin was found in the culture supernatant of four *C. perfringens* types A and one *C. perfringens* type C.

### 2.4. Antimicrobial Susceptibility

**Figure 3:**
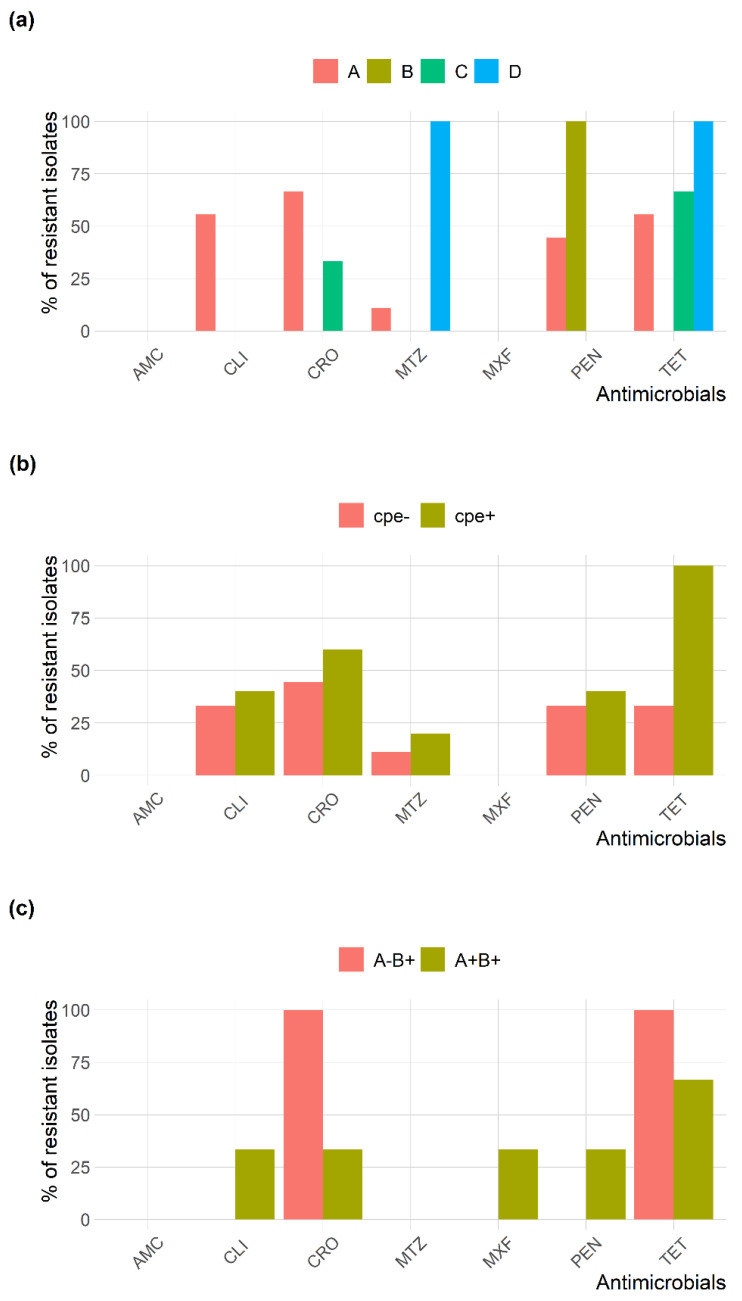
Frequency of antimicrobial resistance of (**a**) *C. perfringens* genotypes, (**b**)* C. perfringens* enterotoxin gene, and (**c**) *C. difficile* genotypes recovered from camel minced meat samples. Antimicrobials are amoxicillin-clavulanate (AMC), clindamycin (CLI), ceftriaxone (CRO), metronidazole (MTZ), moxifloxacin (MXF), penicillin (PEN), and tetracycline (TET).

The distribution of minimum inhibitory concentration (MIC) values of different antimicrobials against the 14 *C. perfringens* and four *C. difficile* isolates recovered from camel minced meat is shown in Figure 2. *C. perfringens* and *C. difficile* isolates had a high frequency of tetracycline (TET) resistance (56% and 75%, respectively), followed by ceftriaxone (CRO) resistance (50% and 50%, respectively). All *C. perfringens* isolates were amoxicillin-clavulanate (AMC) and moxifloxacin (MXF) sensitive; however, all *C. difficile* isolates were only AMC sensitive. The frequency of antimicrobial resistance of *C. perfringens* and *C. difficile* genotypes isolated from camel minced meat is shown in Figure 3. Multidrug resistance was observed in three (21.4%) *C. perfringens* and one (25%) *C. difficile* isolates. The mean multiple antibiotic resistance (MAR) index for resistant *C. perfringens* isolates was 0.30 (range 0.14–0.57), and *C. difficile* isolates was 0.29 (range 0.14–0.43) (Table 3).

## 3. Discussion

Foodborne pathogens cause a wide range of diseases, with serious consequences for human health and the economy. *C. perfringens* is one of the most common foodborne pathogens that contaminate many types of retail meat products and cause food poisoning in humans and domestic animals [22]. Several studies have investigated the occurrence and genotypes of *C. perfringens* in retail food, including chicken [23], beef [24], and sheep [25] meats. However, few studies have determined *C. perfringens* genotypes and antimicrobial resistance in camel meat [26,27]. 

In the present study, *C. perfringens* was isolated from 14% of the tested minced meat samples. This finding is consistent with previous studies that reported the occurrence of *C. perfringens* in 33.7% [27] and 2.7% [26] of tested camel meat samples and swabs, respectively. On the other hand, previous studies have reported the occurrence of *C. perfringens* in 18% of tested minced meat in Turkey [28]; 21.2% of beef slaughtering and butchering processes in China [29]; 44.3% of goat, sheep, and cattle meat in Pakistan [30]; and 23.5% of raw processed meat in South Africa [31]. 

*Clostridium perfringens* types A, B, C, and D have been isolated in this study and type A (64.3%) was the most prevalent, followed by type C (21.5%). Several studies that investigated the distribution of *C. perfringens* types in retail food samples have reported that *C. perfringens* type A was the most common among the types recovered from camel [26,27], beef [24] and sheep [25] meats. However, a recent study in Korea detected only *C. perfringens* type A (33%) in tested chicken and beef meat samples [32]. In another study conducted in sheep meat, *C. perfringens* type A was not detected, and types B, C, and D were isolated [33]. All types of *C. perfringens* are commensal in the gastrointestinal tract of animals, therefore, contamination during slaughter and butchering could be the primary source of *C. perfringens* in meats [34,35].

Toxin genes (*cpa*, *cpb*, *cpe*, and *etx*) were found on the *C. perfringens* isolates recovered in our study. Although all *C. perfringens* isolates were found to be associated with *cpa^+^* gene, only five (35.7%) isolates were associated with the enterotoxins (*cpe^+^*) gene, which is responsible for nearly all *C. perfringens* food poisoning outbreaks [25,36,37]. Similarly, previous studies found that all *C. perfringens* isolates from meat samples were positive for *cap^+^* gene [38,39], suggesting that it might be a universal gene in *C. perfringens* isolated from meat samples [40]. Moreover, the *cpe^+^* gene has been detected in 1.4% [25], 5% [41], and 27.2% [42] of *C. perfringens* isolated from raw and processed meats. In contrast, a recent study did not find *cpe^+^* gene in any of the *C. perfringens* isolates recovered from meat samples in Korea [32]. 

*Clostridium difficile* infections have been increased globally in the last two decades, causing severe intestinal infections in humans [43,44]. Thus, the occurrence of *C. difficile* has been detected in 4% of camel minced meat used in this study, and all isolates were identified as toxigenic. This finding is consistent with previous studies that isolated *C. difficile* from 1.9% of ground beef samples in France [45], 5% of ground beef and hamburger samples in Sweden [46], and 6.3% of lamb meat samples in the Netherlands [47]. However, *C. difficile* was not detected in ground beef in Switzerland [48] and Austria [49]. Food animals are known carriers of *C. difficile* [50], and multiple reports show *C. difficile* shedding in animals at slaughter [51,52]. Contamination of meats may occur due to gut content leakage during evisceration or due to the accumulation of spores within the slaughterhouse environment [53]. The discovery of genetically identical *C. difficile* strains in food, livestock, and humans has raised awareness for *C. difficile* potential as an unspecific foodborne agent [54,55].

ELISA can detect *C. perfringens* and *C. difficile* toxins. In the current investigation, neither *C. perfringens* nor *C. difficile* toxins were found in camel minced meat. In comparison, *C. perfringens* toxins were found in 13% of minced meat samples in Turkey [28]. Although *C. perfringens* and *C. difficile* were isolated from some samples, they were toxin negative, which could be related to the fact that some clostridia are incapable of making toxins [25] or the toxin concentration in the samples may be below the ELISA detection limit (5 ng/mL). 

In Saudi Arabia, antimicrobials have been used in livestock to promote growth and prevent several infectious diseases. However, their use has paradoxically increased the bacterial resistance to antimicrobials [56,57]. In this study, *C. perfringens* and *C. difficile* isolates exhibited a high resistance rate to TET (56% and 75%, respectively). However, all isolates were susceptible to AMC. Similarly, previous reports have shown that *C. perfringens* and *C. difficile* isolates are highly resistant to TET [39,57,58] and are susceptible to beta-lactams [58,59]. In South Korea, more than 93% of *C. perfringens* isolates from beef, poultry, duck, and pork meats were tetracycline resistant [60]. Furthermore, many investigations have found that beta-lactam antibiotics, such as ampicillin, can inhibit *C. perfringens* isolated from beef, poultry, and pork meats from the United States, Belgium, Scandinavia, and India [61,62]. 

Multidrug resistance has been reported in *C. perfringens* [62,63] and *C. difficile* [64,65] isolates from retail meats. In the present study, 21.4% of *C. perfringens* and 25% of *C. difficile* isolates from camel minced meat showed multidrug resistance, higher than the prevalence of multidrug-resistant observed in other studies [11,32,61]. Overuse of antimicrobials (over-the-counter antibiotics without a prescription), use of TET as a growth promoter, and extensive international travel to Saudi Arabia [66] are all possible explanations for the high resistance rate found in this study. In addition, the prevalence and/or emergence of multidrug-resistant *C. perfringens* has been increasing and poses a tremendous public health concern [67]. A recent study has reported that multidrug resistance bacteria in retail meats originates primarily in veterinary healthcare settings or on farms where animals are administered antibiotics in their feeds or treat diseases [68].

The limitation of this study is the small number of minced meat samples collected. However, the collected samples represent only one district and do not represent different regions of Saudi Arabia. Despite this limitation, the study identified that camel meat was contaminated with *C. perfringens* and *C. difficile* and further studies are warranted to determine their prevalence and zoonotic potential.

## 4. Materials and Methods

### 4.1. Sample Collection

A total of 100 camel minced meat samples were randomly collected from small butcher’s shops (n = 50) and supermarkets (n = 50) in Al-Ahsa Governorate, Saudi Arabia, from September 2019 to June 2020. Approximately 100 gm of minced meat was collected aseptically in sterile plastic bags and stored at 4 °C until processing within 12–24 h. For sample processing, 25 gm of each sample was aseptically placed in a sterile plastic bag containing 225 mL sterile peptone solution (Oxoid, Basingstoke, Hampshire, UK) and homogenized using a stomacher at low speed for 3 min. 

### 4.2. Isolation and Counting of C. perfringens

A plate count of viable *C. perfringens* was performed according to Rhodehamel and Harmon [10]. Briefly, homogenized meat samples were serially diluted (10^−1^ to 10^−6^) using a sterile peptone diluent. Dilutions were thoroughly mixed by gentle shaking before each transfer. An aliquot of 0.1 mL of each dilution was inoculated into Tryptose-sulfite-cycloserine (TSC) agar (HiMedia, Mumbai, India) containing egg yolk emulsion (Oxoid, Basingstoke, Hampshire, UK), then spread over agar surface with sterile glass rod spreader. All plates were incubated at 37 °C under anaerobic conditions for 24 h. Plates showing 20–200 black colonies surrounded by white zone were selected for counting. 

For isolation, five suspected colonies from each plate were selected and inoculated into a freshly prepared thioglycollate broth, then incubated under anaerobic conditions at 37 °C for 24 h. For each sample, a loopful was then sub-cultured into 5% sheep blood agar and incubated anaerobically for purification. Colonies were identified based on Gram staining and hemolysis on blood agar. Colonies showing Gram-positive rods and hemolysis on agar were selected for further automated biochemical identification by VITEK-2 compact system using VITEK-2 ANC card for Anaerobes (BioMerieux, Marcyl’Etoile, France). A reference strain of *C. perfringens* ATCC 19574 was used as a control. Purified isolates were sub-cultured into fluid thioglycollate broth and incubated for 24 h under anaerobic conditions to further detect *C. perfringens* toxins in the culture supernatant.

### 4.3. Isolation of C. difficile

The broth enrichment method was used to isolate *C. difficile* [69]. Briefly, 25 gm of minced meat was thoroughly homogenized with 25 mL phosphate-buffered saline (pH 7.4) in a sterile container. One mL of the homogenate was inoculated into 9 mL *C. difficile* moxalactam and norfloxacin broth (CDMN) (Oxoid, Basingstoke, Hampshire, UK), then incubated under anaerobic conditions at 37 °C for 48 h. An aliquot of the broth was subjected to alcohol shock by adding an equal volume of anhydrous ethanol for 1 h in a sterile tube. The tubes were then centrifuged at 1900 *g* for 10 min. Pellets were inoculated into CDMN agar and incubated anaerobically at 37 °C for 48 h. Five suspicious colonies were sub-cultured onto 5% sheep blood agar for purification. Isolates were identified based on Gram staining, colony morphology, and production of proline aminopeptidase. Further biochemical identification was performed by VITEK-2 compact system using VITEK-2 ANC card for Anaerobes (BioMerieux, Marcyl’Etoile, France). A reference strain of *C. difficile* ATCC 43596 was used as a control. Recovered isolates were sub-cultured in TY medium (3% w/v tryptose, 2% w/v yeast extract, and 0.1% w/v thioglycollate) for 48 h at 37 °C for detection of *C. difficile* toxins.

### 4.4. Molecular Identification and Genotyping

Genomic DND was extracted and purified from all *C. perfringens* and *C. difficile* isolates using QIAamp DNA mini kit (Qiagen, Courtaboeuf, France) according to the manufacturer’s instructions.

#### 4.4.1. 16S rRNA Gene Amplification and Sequencing

The extracted DNA (2 μL) was amplified in 20 μL of the final volume of 2X HotStartTaq^®^ Plus Master Mix Kit (QIAGEN, Germantown, MD, USA) and 0.5 mm of each forward (LPW58, 5′- AGGCCCGGGAACGTATTCAC-′3) and reverse (LPW81, 5′-TGGCG AACGGGTGAGTAA-′3) primers. Thermo-cycling conditions were performed in Bio-Rad iCycler Thermal Cycler (Bio-Rad Laboratories, Hercules, CA, USA) as described by Woo, et al. [70]. PCR products were purified by the QIA quick PCR purification kit (Qiagen, Courtaboeuf, France). Purified products were sequenced using an ABI 3500 Genetic analyzer (Applied Biosystems, Foster City, CA, USA). The 16S rRNA gene sequences were subjected to analysis via the National Center for Biological Information (NCBI) Basic Local Alignment Search Tool (https://blast.ncbi.nlm.nih.gov/Blast.cgi; accessed on 11 March 2021) and have been deposited in the GeneBank with accession numbers (MW725396–MW725401, MW732694, MW732695 and MW785763–MW798269). 

#### 4.4.2. Detection of *C. perfringens* and *C. difficile* Toxin Genes by Real-Time PCR

*C. perfringens* toxin genes including alpha (*cpa*), beta (*cpb*), epsilon (*etx*), and enterotoxin (*cpe*) toxin genes, were amplified by real-time PCR using specific primers and probes previously designed by Gurjar, et al. [71]. The 20 μL uniplex reaction mix containing 8 μL PCR grade water, 4 μL of 5X FastStart DNA Master Plus (Roche Diagnostics, Mannheim, Germany), 1 μL of forward and reverse primers, 1 μL of Taqman hybridization probe, and 5 μL DNA template. Cycling conditions comprised initial denaturation at 95 °C for 10 min followed by 40 cycles of denaturation (95 °C for 30 s) and annealing and extension (55 °C for 1 min) using Light cycler 2.0 (Roche Applied Science, Penzberg, Germany). Negative results were considered when no amplification was recorded or when Ct value was higher than 36 cycle.

*C. difficile* toxins A (*tcdA*) and B (*tcdB*) were amplified using a commercial kit (RealStar *Clostridium difficile* PCR Kit, Altona Diagnostics, Hamburg, Germany) according to the manufacturer’s instructions in Light cycler 2.0. 

### 4.5. Detection of C. perfringens and C. difficile Toxin by ELISA

Minced meat samples were homogenized and centrifuged at 17,096 *g* for 10 min, and the supernatant was filtrated through a sterile 0.45 µm syringe filter in a sterile tube. Sandwich ELISA kits (Multiscreen AgELISA Enterotoxemia, Bio-X Diagnostics, Jemelle, Belgium) were used to detect *C. perfringens* toxins (alpha, beta and epsilon) according to the manufacturer’s instructions. However, for detection of enterotoxin, the overnight growth of *C. perfringens* in cooked meat media was heat-inactivated at 75 °C for 20 min then sub-cultured in modified Duncan and Strong Medium [72] and incubated at 37 °C for 24 h. Cells were removed by centrifugation, and the enterotoxin was detected in the supernatant by a commercial latex test (PET-RPLA Toxin Detection Kit, Oxoid, Basingstoke, Hampshire, UK) as per the manufacturer’s instructions. Commercial ELISA Kits (Ridascreen *Clostridium difficile* Toxin A/B, R Biopharm AG, Germany) were used to detect *C. difficile* toxin A/B following the manufacturer’s guidelines.

### 4.6. Antimicrobial Susceptibility Testing

The MIC was determined by broth microdilution methods. Seven different antimicrobials (penicillin (PEN, ≥2 μg/mL), amoxicillin-clavulanate (AMC, ≥16 μg/mL), ceftriaxone (CRO, ≥64 μg/mL), moxifloxacin (MXF, ≥8 μg/mL), clindamycin (CLI, ≥8 μg/mL), metronidazole (MTZ, ≥32 μg/mL), and tetracycline (TET, ≥16 μg/mL)) from seven different antimicrobial classes were used to assess the antimicrobial susceptibility of both *C. perfringens* and *C. difficile*. Antimicrobial solutions were prepared, and a double-fold dilution in brucella broth (0.125–256 μg/mL) was performed in a sterile microtiter plate. A fresh culture from overnight growth was adjusted to 0.5 McFarland standard (10^6^ CFU/mL) and added to each dilution before being incubated at 37 °C for 48 h under anaerobic conditions. The MIC values were determined according to the Clinical Laboratory Standards Institute [73,74]. Sterile brucella broth and *C. perfringens* ATCC 19574 cultures were included in each run as negative and positive controls to assess the method reliability. The MAR index was calculated (number of antimicrobials that isolate showed resistance/total number of antimicrobials that isolate had been evaluated for susceptibility) [75]. However, multidrug resistance was defined as resistance to at least three antimicrobial classes [76].

### 4.7. Data Analysis 

Collected data were visualized with R software (R Core Team, 2019; version 3.5.3), and the “Complex-Heatmap” R package was used to build heatmap [77]. Phylogenetic analysis was performed using MEGA (version 11) software. Multiple sequence alignments were then performed by ClustalW, and the neighbour-joining method with 1000 bootstrap was used to establish the phylogenetic tree.

## 5. Conclusions

Results of this work show that camel minced meat was contaminated with *C. perfringens* and *C. difficile*. Moreover, our results provide further evidence on the emergence of multidrug-resistant strains. Thus, food safety standards and frequent inspections of abattoirs, small butcher shops, and supermarkets should be enforced. Furthermore, proactive antimicrobial agent control measures should be developed to limit the spread of multidrug-resistant strains.

## Figures and Tables

**Table 1:** The number of *C. perfringens* and *C. difficile* isolates recovered from camel minced meat samples collected from small butcher shops and supermarkets.

Sample Source	No. of Tested Samples	No. (%) of	
		* C. perfringens *	* C. difficile *
Butcher shops	50	10 (71.4)	3 (75)
Supermarkets	50	4 (28.6)	1 (25)
Total	100	14 (14%)	4 (4%)

**Table 2:** Distribution of *C. perfringens* and *C. difficile* types and toxin genes in camel minced meat samples.

*Clostridium* spp.	Type	N	Toxin Gene	No. (%)
*C. perfringens*	A	9	*cpa^+^*	5 (35.7)
			*cpa^+^*, *cpe^+^*	4 (28.6)
	B	1	*cpa^+^*, *cpb^+^*, *etx^+^*	1 (7.1)
	C	3	*cpa^+^*, *cpb^+^*	2 (14.3)
			*cpa^+^*, *cpb^+^*, *cpe^+^*	1 (7.1)
	D	1	*cpa^+^*, *etx^+^*	1 (7.1)
*C. difficile*	A^+^B^+^	3	*tcdA*, *tcdB*	3 (75)
	A^−^B^+^	1	*tcdB*	1 (25)

**Table 3:** The antimicrobial-resistant profiles of *C. perfringens* and *C. difficile* isolates recovered from camel minced meat samples.

Genotype	Source	Accession No.	Resistance Patterns ^1^	MAR Index ^2^
*C. perfringens* type C	Supermarket	MW725399	-	0.00
*C. perfringens* type A	Supermarket	MW785765	PEN	0.14
*C. perfringens* type B	Supermarket	MW785767	PEN	0.14
*C. perfringens* type C	Butcher shop	MW785769	TET	0.14
*C. perfringens* type A	Butcher shop	MW725397	PEN, CLI	0.29
*C. perfringens* type C	Butcher shop	MW725398	CRO, TET	0.29
*C. perfringens* type A	Butcher shop	MW725400	CRO, CLI	0.29
*C. perfringens* type A	Butcher shop	MW725401	PEN, TET	0.29
*C. perfringens* type A	Butcher shop	MW785763	CRO, CLI	0.29
*C. perfringens* type D	Butcher shop	MW785764	MTZ, TET	0.29
*C. perfringens* type A	Butcher shop	MW785770	CRO, TET	0.29
*C. perfringens* type A	Supermarket	MW725396	CRO, CLI, TET	0.43
*C. perfringens* type A	Butcher shop	MW785766	CRO, MTZ, TET	0.43
*C. perfringens* type A	Butcher shop	MW785768	PEN, CRO, CLI, TET	0.57
*C. difficile* type A^+^B^+^	Butcher shop	MW798269	TET	0.14
*C. difficile* type A^+^B^+^	Butcher shop	MW798268	CLI, TET	0.29
*C. difficile* type A^−^B^+^	Supermarket	MW732695	CRO, TET	0.29
*C. difficile* type A^+^B^+^	Butcher shop	MW732694	PEN, CRO, MXF	0.43

^1^ CRO: ceftriaxone; CLI: clindamycin; TET: tetracycline; PEN: penicillin; MTZ: metronidazole; MXF: moxifloxacin. ^2^ MAR: Multiple antibiotic resistance index.

## Data Availability

The data presented in this study are available on request from the corresponding author.
